# Clinical Challenges in Profound Thrombocytopenia Associated With Type 2 Heparin-Induced Thrombocytopenia (HIT2)

**DOI:** 10.7759/cureus.105848

**Published:** 2026-03-25

**Authors:** Brielle Coe, Iman Elkhashab, Carlos A Saldarriaga, Dean Johnson, Brianna Cattelino

**Affiliations:** 1 Emergency Medicine, Bayhealth Hospital, Dover, USA; 2 Surgery, Philadelphia College of Osteopathic Medicine, Philadelphia, USA; 3 Radiation Oncology, Drexel University College of Medicine, Philadelphia, USA

**Keywords:** case report, drug-induced thrombocytopenia, heparin-induced thrombocytopenia, serotonin release assay, severe thrombocytopenia

## Abstract

This case describes an unusually severe presentation of immune-mediated heparin-induced thrombocytopenia type 2 (HIT2), marked by an abrupt and profound decline in platelet count to levels below 2000/µL. Although HIT typically presents with moderate thrombocytopenia and thrombotic complications, such an extreme nadir in the absence of disseminated intravascular coagulation (DIC) or other hematologic disorders is highly atypical. The patient's course reinforces the need to consider HIT even when thrombocytopenia appears disproportionately severe and highlights that appropriate anticoagulation can be initiated safely despite concurrent bleeding, provided the diagnosis is promptly recognized.

A 76-year-old man with chronic kidney disease, diabetes, and cardiovascular disease was admitted with acute respiratory failure, community-acquired pneumonia, and a non-ST-elevation myocardial infarction complicated by complete heart block. He received multiple heparin products, including IV continuous heparin, prophylactic dosing, and heparin flushes for catheter maintenance, as well as piperacillin-tazobactam during his hospitalization. Within 12 hours, he developed mucosal bleeding, widespread purpura, and a sudden drop in platelet count from 93000/µL to below 2000/µL.

Laboratory testing confirmed the diagnosis of HIT2, and alternative diagnoses such as DIC and thrombotic thrombocytopenic purpura were clinically excluded. Treatment included immediate cessation of heparin, initiation of argatroban, high-dose intravenous immunoglobulin, and corticosteroids. The patient’s platelet count improved after the withdrawal of both heparin and piperacillin-tazobactam; however, re-exposure to the antibiotic later provoked another platelet decline, suggesting a secondary drug-induced immune mechanism.

This case illustrates the diagnostic and therapeutic challenges posed by severe thrombocytopenia in HIT, especially when overlapping medication effects and bleeding risks complicate management decisions. It emphasizes the importance of timely recognition and the careful balancing of anticoagulation in patients at risk for both thrombosis and hemorrhage.

## Introduction

Heparin-induced thrombocytopenia (HIT) is a potentially life-threatening complication of heparin therapy, broadly categorized into two types. HIT type 1 is a non-immune, transient, and mild drop in platelet count occurring within the first one to two days of heparin exposure. It is generally self-limited and requires no change in therapy [1]. In contrast, HIT type 2 (HIT2) is an immune-mediated disorder characterized by antibodies directed against platelet factor 4 (PF4) bound to heparin. This immune response leads to platelet activation, widespread thrombin generation, and a paradoxical hypercoagulable state despite thrombocytopenia [2].

While a drop in platelet count is a defining feature of HIT2, thrombocytopenia typically presents with nadirs between 20-100 K/µL or a >50% decline in platelets one to two weeks following the initiation of heparin therapy [1]. Profound thrombocytopenia (<10 K/µL) is rare and introduces a management dilemma: although the degree of thrombocytopenia may prompt concern for bleeding, the underlying pathophysiology is prothrombotic rather than hemorrhagic [1]. This creates a treatment paradox in which anticoagulation is the standard of care for HIT2 but would ordinarily be considered contraindicated when severe thrombocytopenia poses a bleeding risk [2].

We present a diagnostically and therapeutically challenging case of HIT2 in a patient who experienced a precipitous drop in platelet count to <2 K/µL from a normal baseline while receiving both prophylactic and therapeutic heparin. The patient presented with petechiae and purpura in the setting of suspected sepsis, concurrent antibiotic use, and hemodialysis, raising a broad differential diagnosis that included thrombotic microangiopathies, disseminated intravascular coagulation (DIC), and therapy-related thrombocytopenia. However, confirmatory testing (positive anti-platelet factor 4 enzyme-linked immunosorbent assay (PF4 ELISA) and serotonin release assay) established the primary diagnosis of HIT2.

The pivotal challenge in this case was navigating the treatment of severe thrombocytopenia in a hypercoagulable state. This required balancing the instinctive caution to avoid anticoagulation due to bleeding risk with the necessity of counteracting the high risk of thrombosis inherent to HIT, along with an awareness of the negative impact of platelet transfusions when antibodies are directed against platelets, as in HIT [1]. This case highlights key diagnostic pitfalls and outlines an anticoagulation strategy appropriate for managing HIT when it presents unusually low on the thrombocytopenic spectrum.

## Case presentation

In this case, we discuss the diagnostic measures taken to achieve a rapid and accurate diagnosis of severe HIT2, leading to prompt treatment and continuous monitoring of real-time treatment response. We present a 76-year-old man with a medical history of hypothyroidism, type 2 diabetes mellitus, morbid obesity, hypertriglyceridemia, hypertension, and chronic kidney disease stage IIIa who presented to the emergency department with shortness of breath, orthopnea, chest pressure, and hyperglycemia. He was found to be bradycardic, with a heart rate in the 40s. Electrocardiography demonstrated complete heart block with ST-segment changes. Initial troponin was markedly elevated at 10000 ng/L. The patient was diagnosed with non-ST-elevation myocardial infarction (NSTEMI) and acute hypoxemic respiratory failure secondary to acute pulmonary edema in the setting of congestive heart failure.

**Figure 1:**
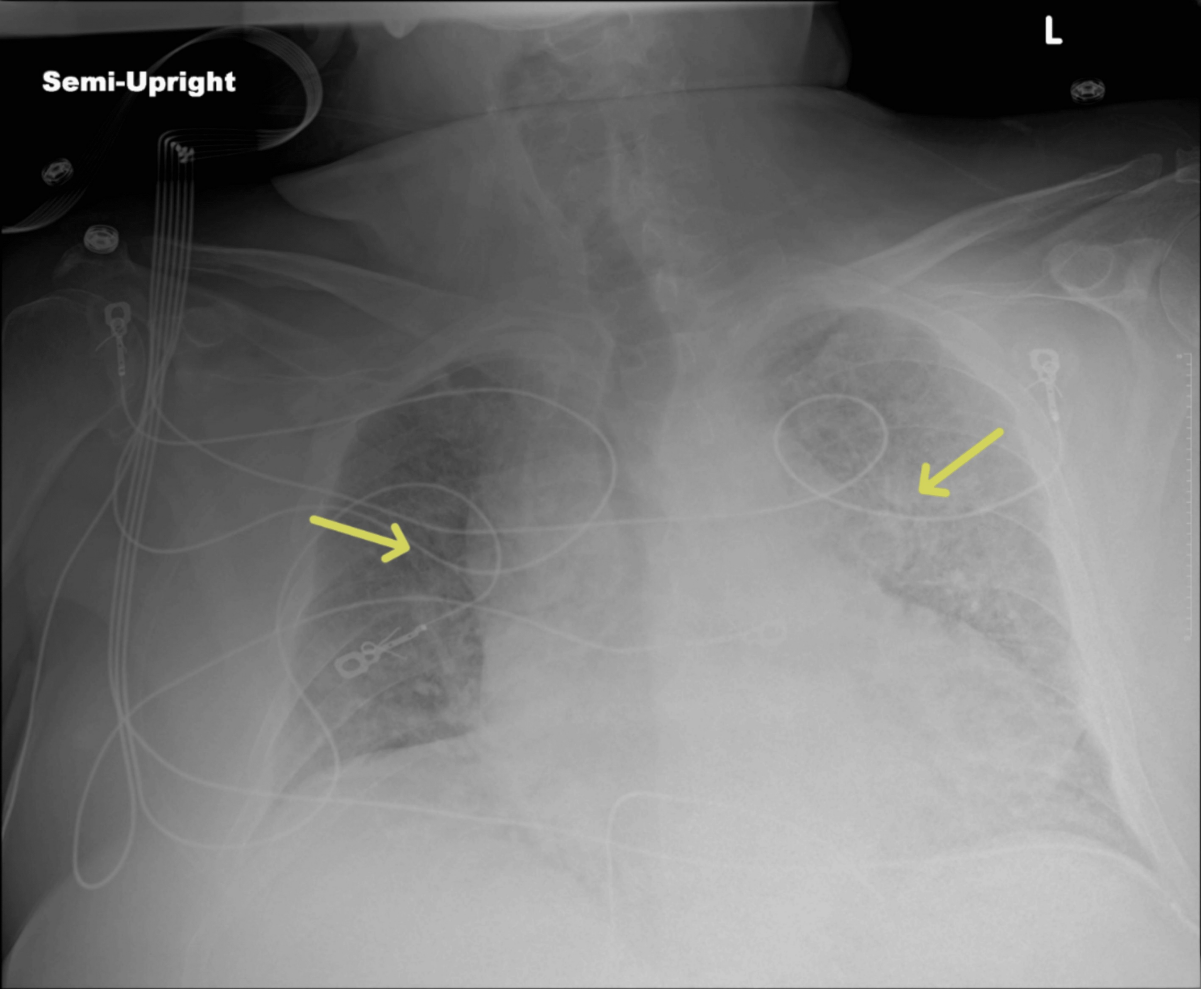
Initial chest radiograph Chest x-ray on admission demonstrated bilateral alveolar opacities and pulmonary congestion (yellow arrows). L: left side

Chest X-ray revealed bilateral alveolar opacities and pulmonary congestion (Figure 1). Transthoracic echocardiography showed a moderately reduced left ventricular ejection fraction with left and right ventricular dilation and a plethoric inferior vena cava. The patient was admitted to the intensive care unit (ICU), where he was intubated for worsening respiratory distress and started on propofol and fentanyl for sedation. Therapeutic anticoagulation with continuous intravenous heparin was initiated on April 7, 2025. On hospital day three (April 9, 2025), he underwent placement of a temporary transvenous pacemaker (TVP) for complete heart block. Acute-on-chronic kidney disease was diagnosed, and intravenous loop diuretics were initiated. He was empirically treated for community-acquired pneumonia with azithromycin, vancomycin, and piperacillin-tazobactam (Zosyn) due to clinical suspicion of CAP, rapidly worsening clinical status, and pending culture results.

During hospitalization, the patient was exposed to multiple sources of heparin, including a continuous infusion (April 7-10), prophylactic subcutaneous heparin (April 11-18), heparin circuit priming during hemodialysis sessions on April 15 and 17, and intermittent heparin flushes through dialysis ports. The patient initially presented with NSTEMI, for which continuous heparin infusion was indicated. Once the patient was intubated, the continuous infusion was stopped, and prophylactic dosing was initiated due to the patient’s clinical status. Progressive renal failure with a decline in glomerular filtration rate to <10 mL/min/1.73 m² prompted the decision to initiate hemodialysis.

**Figure 2:**
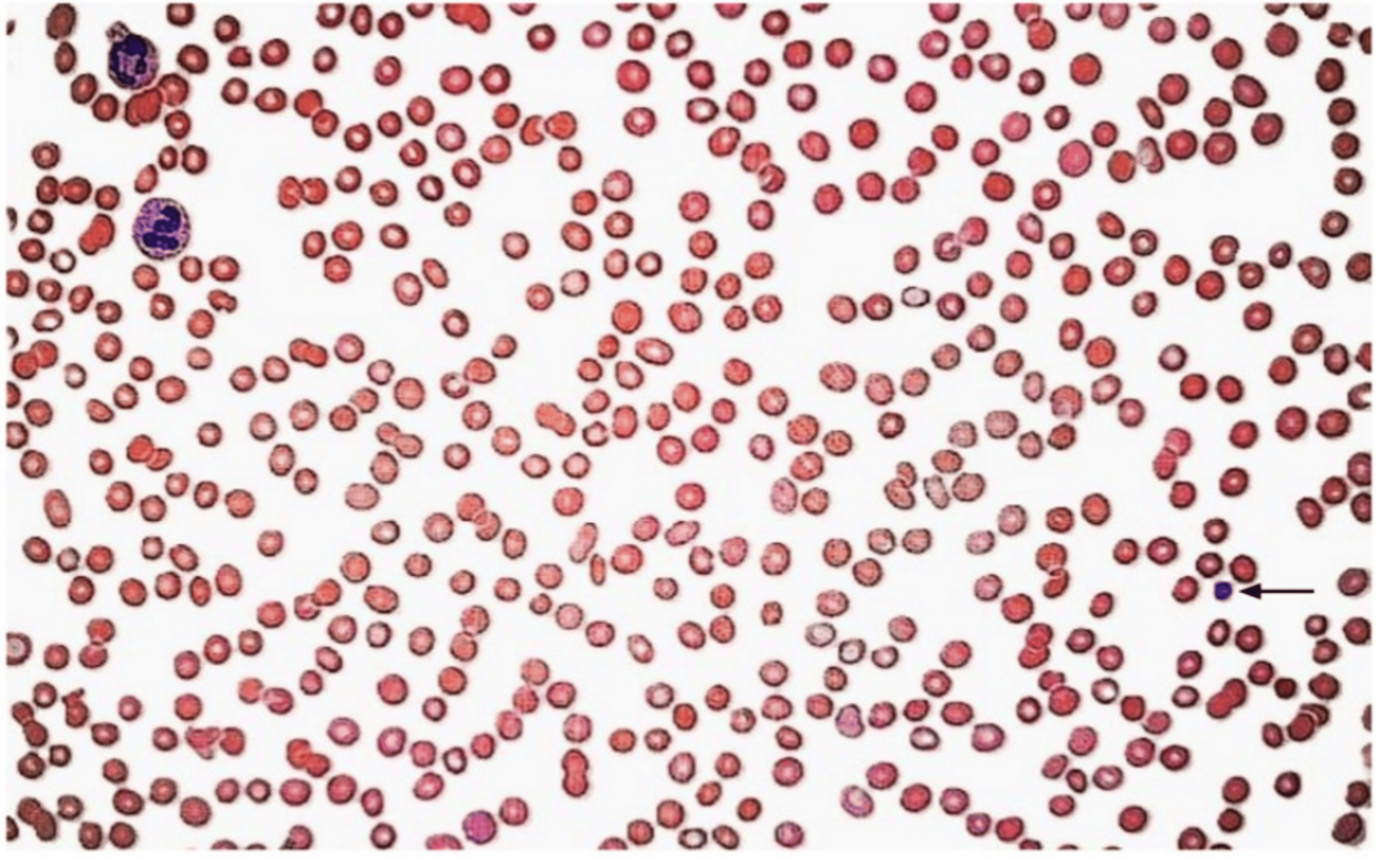
Peripheral blood smear The image demonstrates a normocytic, normochromic red blood cell population with preserved morphology, and a single platelet per high-power field (black arrow). Leukocytes are visible.

On April 17, the patient’s platelet count dropped from 162 K/µL to 93 K/µL. By April 18, the platelet count had declined to <2 K/µL (Figure 2). Physical examination revealed new petechiae and purpura across the torso and upper extremities, as well as mild oropharyngeal bleeding requiring self-suctioning. The patient became somnolent, febrile to 100.6°F, and desaturated to the mid-80s on bilevel positive airway pressure (BiPAP) support after being extubated on April 16.

Laboratory evaluation on April 18 also revealed a fibrinogen level of 650 mg/dL, D-dimer of 8689 ng/mL, prothrombin time (PT) of 17.7 seconds, international normalized ratio (INR) of 1.55, partial thromboplastin time (PTT) of 37 seconds, lactate dehydrogenase (LDH) of 370 U/L, haptoglobin of 434 mg/dL, and a corrected reticulocyte count of 1.2%. Direct antiglobulin testing was positive for C3b/C3d and negative for IgG. The PF4 antibody ELISA returned positive. Head CT and computed tomographic angiography (CTA) of the head and chest ruled out acute intracranial hemorrhage or large-vessel thromboembolism but showed bilateral pulmonary opacities and significant calcified atherosclerotic disease of the carotid arteries. Doppler ultrasound of the lower extremities was negative for deep vein thrombosis, while arterial Doppler studies demonstrated monophasic waveforms suggestive of downstream arterial stenosis or occlusion attributable to atherosclerotic disease.

**Figure 3:**
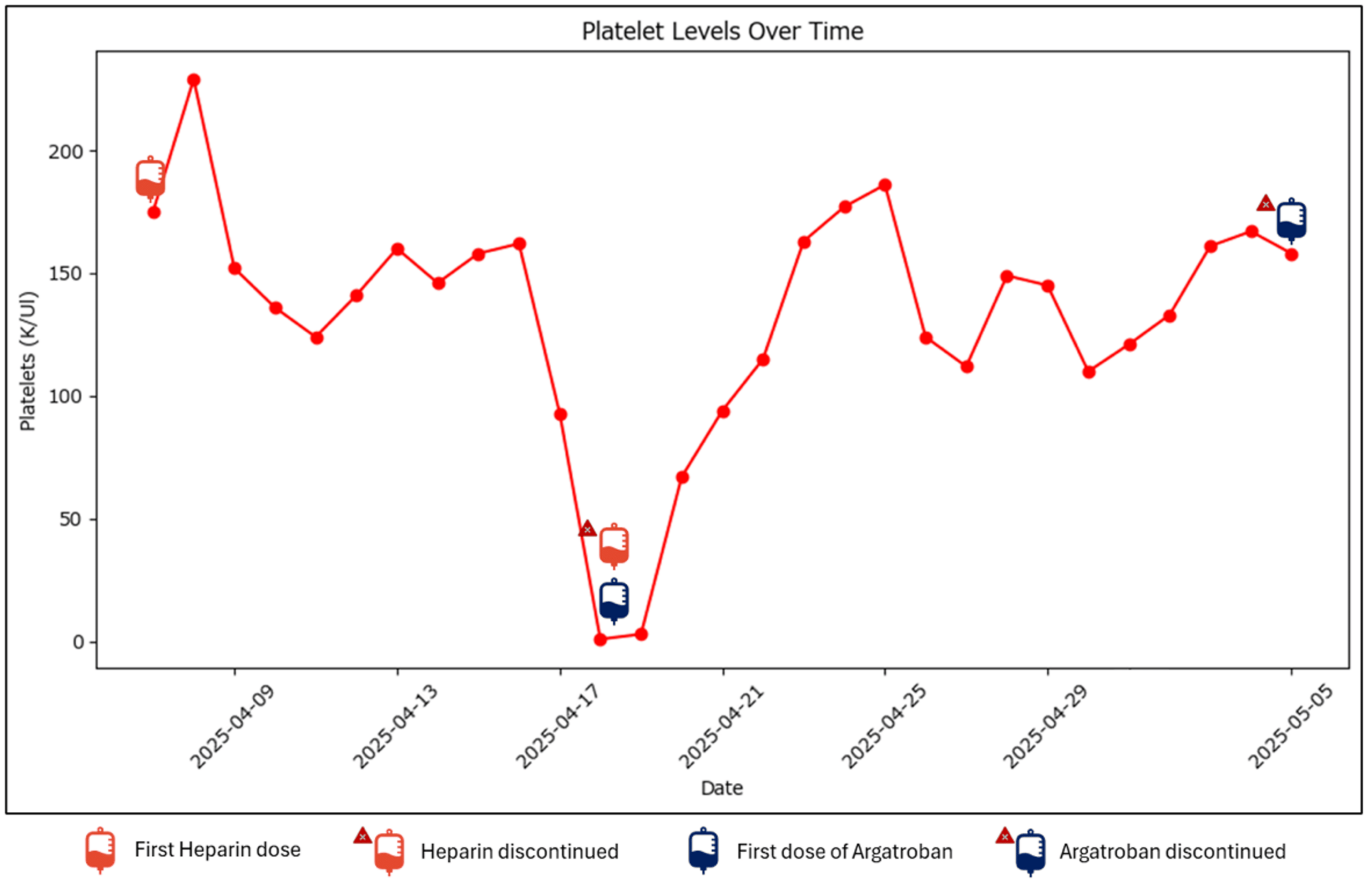
Platelet levels over time in relation to anticoagulation therapy The graph demonstrates a temporal association between heparin administration and a marked decline in platelet count. The patient received the first dose of heparin on April 8, 2025, followed by a steady decline in platelet levels, reaching a nadir on April 17, 2025. Heparin was discontinued on the same day argatroban, a direct thrombin inhibitor, was initiated. Following this transition, platelet counts began to recover, with a sustained upward trend observed over the subsequent days. Argatroban was discontinued on May 4, 2025, once platelet levels had normalized. The timeline supports a diagnosis of HIT, given the characteristic drop in platelets after heparin initiation and improvement after discontinuation and initiation of non-heparin anticoagulation. HIT: heparin-induced thrombocytopenia

**Figure 4:**
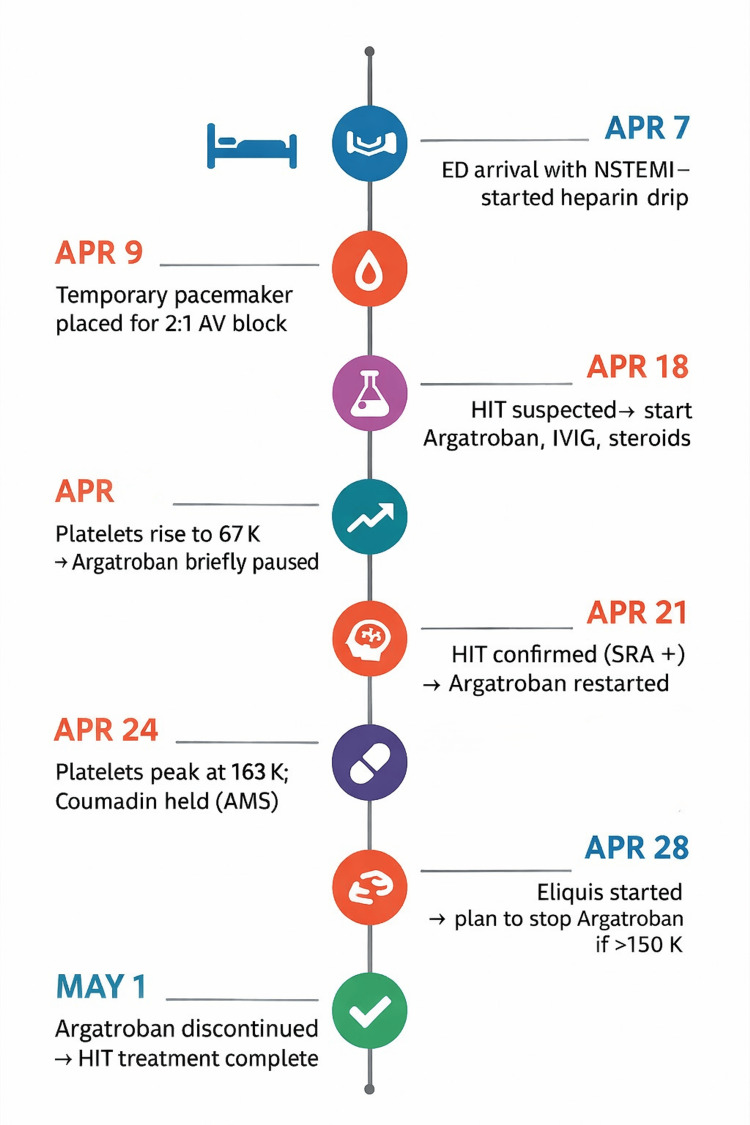
Clinical timeline of events from hospital admission to completion of HIT treatment The patient presented on April 7, 2025, with a NSTEMI and was initiated on a continuous heparin infusion. On April 9, 2025, a temporary pacemaker was placed for symptomatic 2:1 AV block. By April 18, a significant decline in platelet count raised clinical suspicion for HIT, prompting discontinuation of heparin and initiation of argatroban, along with IVIG and corticosteroids. On April 19, platelet levels began to recover, reaching 67 K/µL, at which point argatroban was briefly paused due to persistent thrombocytopenia, elevated bleeding risk, and pending diagnostic tests. Time was also allowed to reassess the coagulation profile as the platelet count began to change significantly. HIT was subsequently confirmed by SRA on April 21, and argatroban was restarted. Platelets continued to rise, peaking at 163 K/µL on April 24. On April 28, the patient was transitioned to apixaban (Eliquis) to provide long-term non-heparin anticoagulation, given the patient’s persistent need for anticoagulation due to cardiac conditions. Argatroban was discontinued on May 5, 2025, following completion of HIT treatment to ensure adequate oral anticoagulation for management after discharge. AV: atrioventricular; NSTEMI: non-ST-elevation myocardial infarction; HIT: heparin-induced thrombocytopenia; IVIG: intravenous immunoglobulin; SRA: serotonin release assay; ED: Emergency Department; AMS: altered mental status

**Figure 5:**
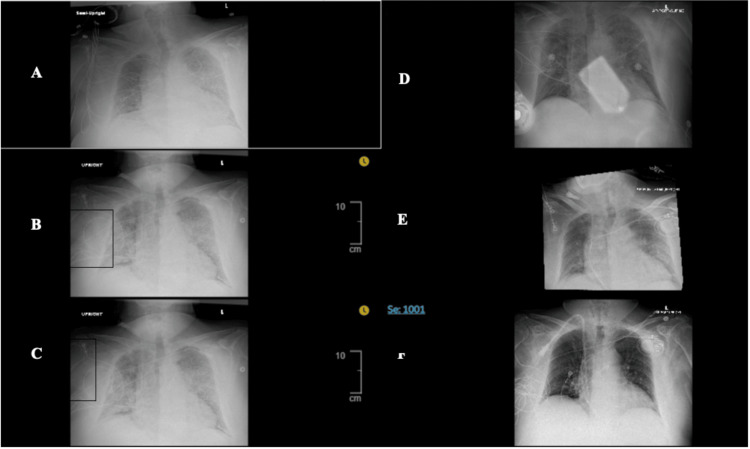
Chest radiographs throughout inpatient stay This panel of images represents the patient’s chest radiographs obtained throughout his admission. These serial radiographs demonstrate stepwise improvement of the patient’s pneumonia, as evidenced by the resolution of the alveolar opacities. The images correspond to the following dates: A (April 18, 2025), B (April 19, 2025), C (April 20, 2025), D (April 21, 2025), E (April 23, 2025), and F (May 12, 2025).

All heparin products were discontinued following clinical suspicion of HIT based on a positive PF4 ELISA. The patient was initiated on argatroban and received two units of platelet transfusions. Intravenous immunoglobulin (IVIG) and high-dose corticosteroids were administered during the same period. These therapies were administered in combination due to severe immune-mediated disease, aiding in the inhibition of new autoantibody production and preventing disease worsening. Argatroban was stopped on April 22, 2025, due to the elevated bleeding risk and marked thrombocytopenia, and was later restarted on April 24, 2025, once platelets reached 163 K/µL. No thromboembolic events occurred, and platelet counts gradually returned to baseline (Figure 3). For reference, Figure 4 and Table 1 provide a clear representation of events along with the initiation and discontinuation of medications immediately surrounding the HIT event. In addition, Figure 5 illustrates the progression of the patient’s chest radiographs over time, which, although tracking the progression of the patient’s pneumonia, further supports the absence of HIT-related thromboembolic events.

**Table 1:** Medication administration timeline This table outlines the timeline and modifications in the administration of anticoagulants and additional medications for our patient immediately before and after the HIT event. The patient completed a full course of high-dose corticosteroids on a four-day regimen and was continued on an argatroban regimen in the days following the HIT2 event. HIT2: heparin-induced thrombocytopenia type 2; IVIG: intravenous immunoglobulin

Medication	Start date	End date
Heparin (IV continuous infusion)	04/07/25	04/10/25
Heparin (subcutaneous prophylaxis)	04/07/25	04/18/25
Heparin (dialysis circuit priming)	04/15/25	04/17/25
Heparin (dialysis port flushes)	04/15/25	04/17/25
Argatroban (initial course)	04/18/25	04/22/25
Argatroban (restarted)	04/24/25	05/05/25
IVIG	04/18/25	04/18/25
High-dose corticosteroids	04/18/25	04/21/25
Platelet transfusions (2 units)	04/18/25	04/18/25

## Discussion

We found that this patient developed an unusually severe form of HIT2. A nadir platelet count of less than 2000/µL was observed, which is highly uncommon in classic HIT presentations. Despite the profound thrombocytopenia, the diagnosis was confirmed through anti-PF4 ELISA and serotonin release assay (SRA). This case was established as confirmed immune-mediated HIT with a noteworthy hematologic presentation. The primary clinical challenge in this case was managing the paradox of a profoundly thrombocytopenic patient in a hypercoagulable state, which required careful diagnostic consideration and a tailored therapeutic approach.

In typical HIT2, platelet counts fall by over 50% but rarely drop below 20000/µL [1]. The thrombocytopenia observed in this patient significantly exceeded that range, raising the possibility of concurrent causes of platelet destruction. The patient was critically ill and exposed to multiple potential contributors to thrombocytopenia, including sepsis, renal failure with uremia, and intermittent hemodialysis. Possible differential diagnoses included dual immune thrombocytopenia (HIT+antibiotic-induced immune thrombocytopenia (DITP)), sepsis-induced consumptive thrombocytopenia, dialyzer-associated platelet destruction, and uremia-induced thrombocytopenia, each of which could have contributed to the severity of the thrombocytopenia [2,3]. However, no additional immunologic testing was performed during the event.

The patient did not fulfill the International Society on Thrombosis and Haemostasis (ISTH) diagnostic criteria for overt DIC. These criteria consist of a scoring system based on platelet count, D-dimer or fibrin degradation products, PT, and fibrinogen levels. Although the patient exhibited elevated D-dimer levels and mild PT prolongation, his fibrinogen level remained elevated (650 mg/dL) rather than decreasing as expected. Additionally, his platelet count began to recover following heparin cessation, making consumptive coagulopathy less likely.

Thrombotic thrombocytopenic purpura (TTP) was also considered, particularly due to the abrupt platelet fall and positive complement activation (DAT C3b/C3d positive). However, key diagnostic features of TTP were absent, including microangiopathic hemolytic anemia (normal LDH, haptoglobin, and reticulocyte count). The absence of schistocytes on peripheral smear further supports the exclusion of TTP [4]. A disintegrin and metalloproteinase with thrombospondin type 1 motif, member 13 (ADAMTS13) activity was mildly reduced (0.54), but not severely deficient (<10%), further ruling out classic TTP. Levels of ADAMTS13 activity ranging from 10% to 50%, defined as partial ADAMTS13 deficiency, have been shown to be sufficient to prevent TTP, whereas levels below 10% are a hallmark finding of TTP [5]. Additionally, partial deficiency rarely causes severe symptoms.

Despite confounding factors, the patient's laboratory findings and temporal relationship to heparin exposure supported a diagnosis of HIT as the dominant etiology. The 4T score, which aids in distinguishing HIT from other causes of thrombocytopenia, was seven out of eight based on the criteria. This suggests a high probability of HIT compared to other etiologies. Confirmation of the diagnosis via SRA and other laboratory testing was particularly valuable in ruling out non-immune causes of thrombocytopenia, such as TTP and DIC. Compared to previous case reports, in which thrombocytopenia in HIT tends to remain moderate (20 K/µL-100 K/µL), and bleeding is rare, this case is notable for its extreme platelet nadir in the absence of DIC and in the presence of mucocutaneous bleeding [1,6]. This is one of the few cases in the literature documenting HIT with a platelet count below 2 K/µL confirmed by SRA, requiring active bleeding management.

Therapeutically, this patient was managed with discontinuation of heparin and other potential offending medications, initiation of argatroban, and administration of IVIG, high-dose corticosteroids, and two units of platelet transfusions due to increased bleeding risk. Although platelet transfusion is generally avoided in HIT because of potential immune activation and increased thrombosis risk, it is considered acceptable in cases of severe thrombocytopenia [7]. The combined immunomodulatory approach may have contributed to the gradual platelet recovery, which began approximately four days after heparin cessation. In typical HIT2 cases, uninterrupted anticoagulation is critical to prevent thrombosis. In contrast, the severity of thrombocytopenia in this patient necessitated a temporary hold on argatroban after April 19, 2025; it was restarted after HIT2 was confirmed via SRA testing on April 21, 2025. The decision to initiate anticoagulation with argatroban despite severe thrombocytopenia aligns with evidence-based recommendations emphasizing thrombosis prevention over bleeding risk in HIT [2]. IVIG has been associated with rapid platelet recovery, with studies demonstrating a 50-100% increase within five days of administration [2]. In this case, platelet counts rose progressively following IVIG and steroid therapy, allowing safe reinitiation of anticoagulation. Although not outlined in current guidelines, steroid management was used simultaneously to aid in the recovery of immune-mediated thrombocytopenia. This may aid in inhibiting the immune response, promoting recovery in this highly unusual and severe circumstance. 

A key strength of this case is the comprehensive diagnostic workup, including confirmatory testing, which supported the primary diagnosis despite atypical features. Additionally, the therapeutic approach highlights real-world decision-making in managing a critically ill patient with opposing risk factors and evolving clinical needs. A significant limitation is the lack of additional immune testing to fully rule out dual immune HIT or other immune-mediated cytopenias, which could have strengthened the differential assessment. Nevertheless, the clinical course and response to therapy support the predominance of HIT in this patient’s presentation.

The implications of this case are relevant for both clinicians and researchers. It illustrates that extreme thrombocytopenia does not exclude HIT and that confirmatory testing is essential in ambiguous presentations. Moreover, it reinforces the principle that anticoagulation should not be withheld solely because of thrombocytopenia when HIT is strongly suspected. However, special consideration should be given to timing and dosing. Future research should explore the immunologic underpinnings of atypical HIT presentations, including whether early immunosuppressive therapies such as IVIG or corticosteroids can mitigate platelet destruction and accelerate recovery.

## Conclusions

In conclusion, this case underscores a rare and diagnostically challenging presentation of HIT2, marked by profound thrombocytopenia and mucosal bleeding, both of which are atypical in HIT. The case emphasizes the importance of maintaining a high index of suspicion, using confirmatory testing, and balancing bleeding risk with the necessity of anticoagulation. It adds to the limited literature describing extreme thrombocytopenic presentations of HIT and provides insight into successful clinical strategies for managing this paradoxical and potentially fatal condition.
